# Heterogeneity of Locked‐Pasture Snow Conditions Modulate Habitat and Movement Choices of a Facultative Migrant

**DOI:** 10.1002/ece3.70925

**Published:** 2025-02-22

**Authors:** Katherine B. Gura, Glen E. Liston, Adele K. Reinking, Bryan Bedrosian, Kelly Elder, Anna D. Chalfoun

**Affiliations:** ^1^ Wyoming Cooperative Fish and Wildlife Research Unit, Department of Zoology and Physiology University of Wyoming Laramie Wyoming USA; ^2^ Program in Ecology and Evolution University of Wyoming Laramie Wyoming USA; ^3^ Cooperative Institute for Research in the Atmosphere Colorado State University Fort Collins Colorado USA; ^4^ Teton Raptor Center Wilson Wyoming USA; ^5^ Graduate Degree Program in Ecology Colorado State University Fort Collins Colorado USA; ^6^ Department of Fish Wildlife, and Conservation Biology Fort Collins Colorado USA; ^7^ US Forest Service Rocky Mountain Research Station Fort Collins Colorado USA; ^8^ U.S. Geological Survey Wyoming Cooperative Fish and Wildlife Research Unit, Department of Zoology and Physiology University of Wyoming Laramie Wyoming USA

**Keywords:** facultative, habitat selection, ice crust, migration, movement behavior, plasticity, snow, winter

## Abstract

Habitat selection and movement are key mechanisms by which animals can respond to and potentially cope with highly variable environmental conditions. Optimal responses likely vary, however, depending on the severity and scope of conditions. We tested this hypothesis using a facultative migrant species, the Great Gray Owl ( *Strix nebulosa* ), which exhibits high inter‐ and intra‐individual variation in the timing, direction, and distance of winter movements. Specifically, we evaluated whether episodic, spatiotemporally variable “locked‐pasture” snow conditions, which restrict access to subnivean food, prompted shifts in habitat selection or long‐distance movements by owls. We quantified the movement of 42 owls using global positioning system (GPS) data within the Greater Yellowstone Ecosystem, USA, during 2017–2022. We used a novel ecological application of SnowModel, a snow evolution modeling system, to estimate fine‐scale, physical snow properties likely to influence access to prey. Variables included snow depth, snow crusts produced by wind, and ice crusts produced by melt‐freeze and rain‐on‐snow events. Owls avoided heterogeneously distributed wind crusts via local shifts in habitat selection. More homogenous ice crusts elicited long‐distance movements away from affected home ranges. Finally, owls employed both proximate shifts in habitat selection and long‐distance movements to avoid deeper snow. Ultimately, owls exhibited behavioral flexibility in response to limiting snow conditions that can vary in terms of severity, spatial extent, and duration. Such behavioral responses determine species distribution, with implications for population and community dynamics in spatiotemporally variable systems. Understanding the effects of, and responses to, environmental controls is increasingly important given the scope of on‐going global change.

## Graphical abstract

We evaluated how the spatiotemporal dynamics of rapidly‐changing environmental conditions influence facultative movement behavior, using the Great Gray Owl (*Strix nebulosa*) in the Greater Yellowstone Ecosystem as a model system. Owls avoided episodic “locked‐pasture” snow conditions, which restrict access to subnivean prey, but the specific behavioral strategies varied according to the severity, spatial extent, and duration of limiting snow conditions, with spatially‐variable wind crusts prompting shifts in proximate habitat selection, and more widely distributed ice crusts eliciting long‐distance movements. We demonstrate that behavioral plasticity is key for coping with unpredictable conditions, the spatiotemporal scales of which underlie facultative movements and resultant species distribution and community dynamics in changing world Photos by Steve Mattheis.

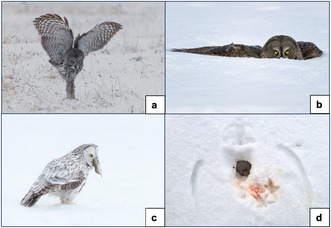

## 1 Introduction

The global climate system is changing at unprecedented rates and scales, resulting in increasingly variable and extreme environmental conditions (IPCC 2023; Seneviratne et al. 2021). Behavior is key for understanding the proximate, near‐term effects of such change on individual organisms (Hollander, Snell‐Rood, and Foster 2015; Snell‐Rood 2013). Indeed, behavioral responses can occur relatively rapidly and are more readily observable compared with other adaptive changes, such as gene frequency shifts associated with natural selection or range shifts associated with population‐level responses (Beever et al. 2017; Chen et al. 2011). The extent to which individuals exhibit behavioral flexibility, therefore, can be an important indicator of the susceptibility of populations to increasingly unpredictable or severe conditions (Berger‐Tal et al. 2011). Although knowledge of behavioral responses to directional, chronic change is growing (e.g., Davidson et al. 2020; Hall and Chalfoun 2018; Tøttrup et al. 2008), how increased environmental variability and acute stressors influence key behaviors is largely unknown for most organisms (Beever et al. 2017).

Proximate habitat shifts (Chalfoun and Martin 2010; Shipley et al. 2019; Wolff et al. 2020) and long‐distance movements (Alerstam and Hedenström 1998; Dingle 1996; Newton 2008; Robinson et al. 2009) are two behavioral tactics that organisms employ to cope with environmental stressors. Each strategy may be advantageous, depending, in part, on the availability and distribution of resources (Bastille‐Rousseau et al. 2017; Harel et al. 2016; Morris 1992). The degree of plasticity in movement behavior likely is also a primary mechanism by which labile organisms cope with spatiotemporally variable, changing conditions (Tøttrup et al. 2008). Facultative migrants (i.e., animals that migrate intermittently in response to experienced conditions) are assumed to be particularly plastic and well‐adapted for systems with lower spatiotemporal predictability (Hahn et al. 2004; Newton 2006, 2012), although the mechanisms underlying facultative movements remain poorly understood. Understanding the contexts under which proximate habitat shifts versus long‐distance movements manifest remains critical for identifying the underlying mechanisms and evolutionary basis of movement strategies (Newton 2006, 2012; Shaw 2016), and for determining species' resilience and the distribution of organisms, especially in a rapidly changing world.

Snow is a primary determinant of environmental conditions, particularly in mid‐latitude, high‐elevation, and Arctic systems where snow persists for a majority of the annual cycle (Bokhorst et al. 2016). Considering the susceptibility of snow regimes to climatic change (Gulev et al. 2021; Hansen et al. 2010; Liston and Hiemstra 2011) and the key role that snow plays in ecological processes, there is a strong impetus to understand how increasingly variable snow conditions impact organisms. For example, the phenomenon of “locked pastures” (Hansen et al. 2013), wherein food resources become unavailable because of restrictive snow or ice conditions (Forchhammer and Boertmann 1993; Helle 1984), can occur episodically and is increasing in frequency and severity because of changing climatic conditions (Hansen et al. 2014; Peeters et al. 2019; Serreze et al. 2021).

In general, some organisms appear restricted in their capacity to cope with unpredictable, limiting snow conditions (e.g., icing events; Hansen et al. 2013; Riseth, Tømmervik, and Bjerke 2016), whereas for other species, the ability to respond adaptively may be context‐dependent (e.g., spring snowstorms, icing events; Hahn et al. 2004; Hansen et al. 2019). Negative effects of ice‐locked forage have been documented for several Arctic herbivore species (Berger et al. 2018; Kausrud et al. 2008; Putkonen et al. 2009; Stien, Ims, and Albon 2012; Tyler 2010), although the potential impacts on other taxa (e.g., carnivores) remain relatively unknown. Locked pastures generally are associated with severe rain‐on‐snow icing events (Bintanja 2018), yet the effects of other snowpack properties that can reduce access to forage, including melt‐freeze cycles (Pedersen et al. 2015; Peeters et al. 2019), wind‐produced snow crusts (Li and Pomeroy 1997), and deep snow (Pedersen et al. 2015), are less understood. Hence, an improved understanding of behavioral responses to limiting snow conditions, and the underlying mechanisms and spatiotemporal scales at which responses manifest, is warranted.

**FIGURE 1:**
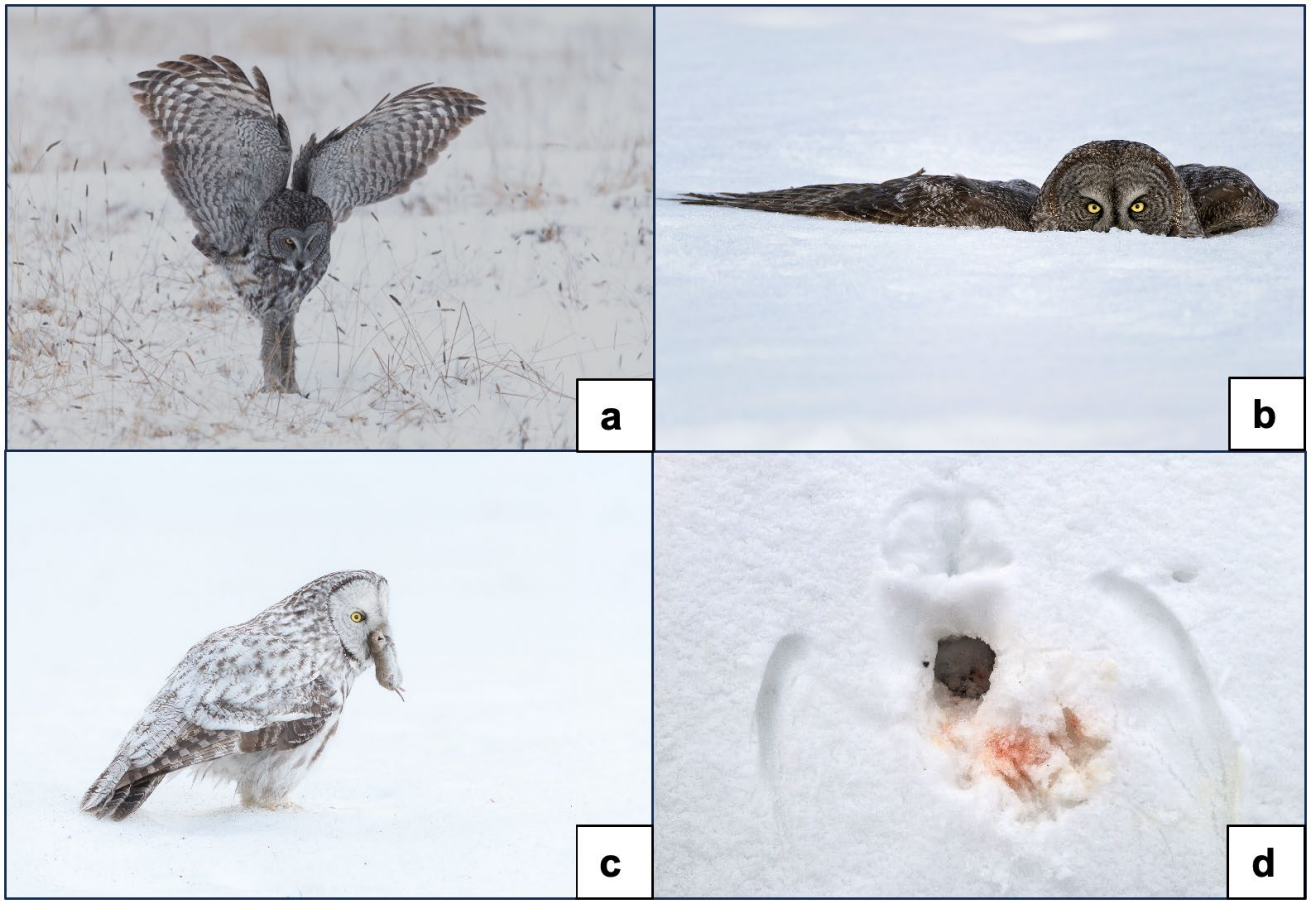
Great Gray Owls hunting for subnivean small mammals (a–c) and an owl print with blood (d), indicating a successful foraging attempt. (Photos by Steve Mattheis).

We evaluated how variable, limiting environmental conditions influence proximate habitat choices versus broader, long‐distance movements, using a facultative migrant population of Great Gray Owls ( *Strix nebulosa* ) as a focal system. The Great Gray Owl is a holarctic raptor that preys on small mammals, including Northern Pocket Gophers ( *Thomomys talpoides* ) and vole (*Microtus*) species (Nero 1980; Gura 2023 ). During winter, Great Gray Owls predate subnivean small mammals (Clark, Duncan, and Doughtery 2022; Nero 1980; Figure 1) by employing a perch‐and‐pounce hunting strategy: they perch, often in or adjacent to open meadow habitat, and listen for prey moving beneath the snow. They then hover over and drop directly onto the prey, capturing animals through up to 45 cm of snow (Collins 1980). Great Gray Owls therefore may be susceptible to locked‐pasture snow conditions that restrict their ability to capture subnivean prey (Mysterud 2016). Great Gray Owls in the southern portion of their North American range are partially migratory (Bull, Henjum, and Rohweder 1988; Franklin 1988; Gura 2023 ), and observed winter movement patterns include residency, altitudinal and long‐distance movements to discrete winter ranges, and seasonal nomadism (Teitelbaum and Mueller 2019; Watts and Cornelius 2024). The proximate mechanisms underlying such variation in movement behavior remain unknown, although snow conditions likely are a key factor. Moreover, at their southern range extent, Great Gray Owls occupy a highly seasonal, mountainous environment that is spatiotemporally heterogenous, where changing snow conditions may influence facultative movement behavior.

We tested the hypothesis that facultative movement strategies of wild animals are modulated by the severity and spatiotemporal extent of limiting conditions. We predicted that proximate habitat shifts are employed when limiting conditions are spatially heterogeneous and temporally restricted, whereas longer‐distance movements away from home ranges occur in response to spatiotemporally extensive constraints. Specifically, we expected that snow conditions restricting owls' access to subnivean prey, including more severe and persistent snow crusts and increased snow depths, would prompt avoidance responses. We further predicted that when limiting snow conditions were heterogeneously distributed, owls would proximately avoid snow crusts and deeper snow via shifts in fine‐scale habitat selection. Finally, we postulated that owls would initiate longer‐distance movements in response to larger expanses of severe snow crusts and deep snow, and more persistent snow crusts. We tested our predictions using fine‐scale animal movement data and novel, biologically relevant snow information derived from a state‐of‐the‐art snow modeling approach.

## 2 Materials and Methods

### 2.1 Study Area

**FIGURE 2:**
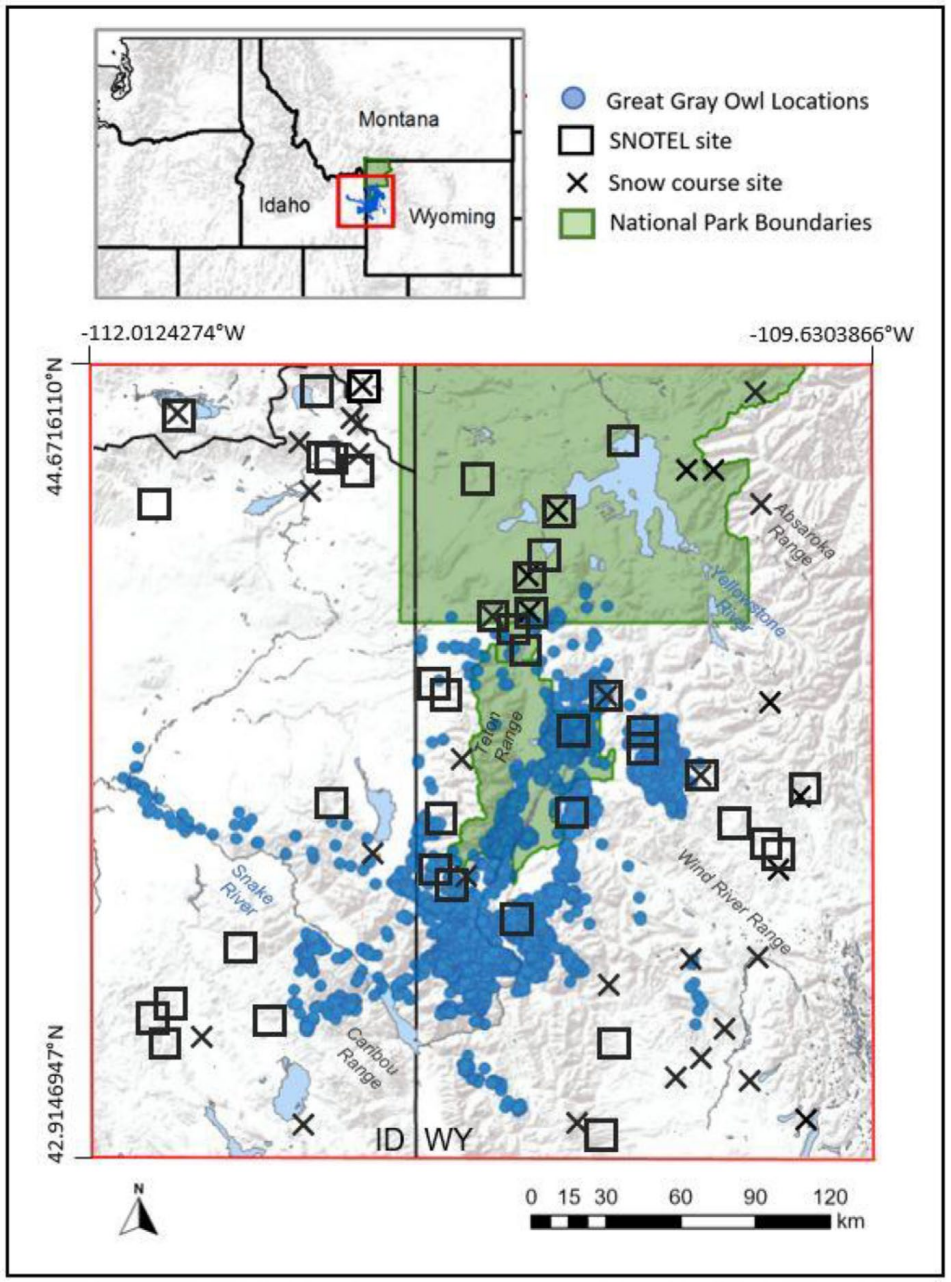
Study area within the Greater Yellowstone Ecosystem in northwestern Wyoming and eastern Idaho. Great Gray Owl winter locations (*n* = 21,113) collected from Global Positioning System transmitters (*n* = 42) during 2017–2022 are shown with blue circles. Locations of Natural Resources Conservation Service Snow Telemetry and snow course sites are shown with black squares and crosses, respectively. Observed snow depth and snow‐water equivalent values from these sites (*n* = 77) were assimilated in the SnowModel simulations.

We conducted our research within the Greater Yellowstone Ecosystem (GYE) in Wyoming, USA, near the southern extent of the Great Gray Owl's contiguous range in North America (Figure 2). The region is topographically variable and contains the rugged Teton Range that extends up to ~4100 m a.s.l. and includes surrounding foothills, riparian corridors, lower‐elevation valleys (~1450–1950 m a.s.l.), and diverse habitat types including forests, sagebrush steppe, montane meadows, wetlands, agricultural zones, and residential areas. Primary tree species include lodgepole pine ( *Pinus contorta* ), aspen ( *Populus tremuloides* ), Douglas fir ( *Pseudotsuga menziesii* ), sub‐alpine fir ( *Abies lasiocarpa* ), cottonwood ( *Populus angustifolia* ), Engelmann spruce ( *Picea engelmannii* ), blue spruce ( *Picea pungens* ), and white‐bark pine ( *Pinus albicaulis* ). Generally, the area is characterized by long‐lasting, cold, snowy winters followed by relatively warm, dry summers. In the valley floor of Teton County, Wyoming during 1991–2020, the average temperature was −9°C during January and 15°C during July, and average precipitation was 71 mm and 31 mm for each month, respectively (High Plains Regional Climate Center 2020).

### 2.2 Owl Data

We captured adult male and female Great Gray Owls during 2017–2021, primarily using pan, bal‐chatri, and dho gazza traps and live mouse lures (Bloom, Clark, and Kidd 2007). We outfitted owls with global positioning system (GPS) remote‐download transmitters (Lotek SwiftFix models PinPoint VHF 240, 1200, and 1800) using either a backpack‐style attachment with tubular Teflon ribbon or a tail‐mount attachment (Bloom, Clark, and Kidd 2007). Transmitters collected GPS locations throughout the annual cycle, with the number of locations per day ranging from 1 to 24, depending on the unit. All required state and federal permits were acquired for this project, and captures were conducted under Wyoming Game and Fish Department Chapter 33 Permit #1011 and United States Geological Survey (USGS) federal bird banding permit #24140.

### 2.3 Snow Data

To evaluate snow conditions in relation to the Great Gray Owl movement, we estimated snow characteristics across the study area and period using SnowModel (Liston and Elder 2006; Liston et al. 2020), a modeling system that simulates snowpack evolution across space and time. We modeled the evolution of snow conditions within our study area from September 1, 2017 to August 31, 2022 at two spatiotemporal resolutions to address our study objectives. First, we simulated snow characteristics at a 3‐h time step and 30 m × 30 m spatial resolution to assess fine‐scale habitat selection in response to local snow conditions. Second, we modeled snow evolution at a 3‐h time step and 500 m × 500 m spatial resolution to assess the probability of long‐distance movements relative to broad‐scale snow conditions.

Inclusion of meteorological, topographical, and land‐cover data enables SnowModel to account for primary, broad‐scale (i.e., synoptic‐scale) controls of snow conditions, such as weather and storm cycles, temperature, wind, presence of forest versus non‐forest habitat, elevation, ridges versus gullies, windward versus leeward aspects, and sun‐exposed versus shaded areas (Daly 2006; O'Neill et al. 1986; Watson et al. 2006). For meteorological forcing, we used National Land Data Assimilation System, Version 2 (NLDAS‐2) meteorological analysis data, which consisted of weather data at 1‐h time steps with 1/8th‐degree spatial resolution (Mitchell et al. 2004; NLDAS 2022; Xia et al. 2012). For topography, we used USGS digital elevation model (DEM) data from the 3D Elevation Program (3DEP) (USGS 2020). To quantify land‐cover type, we used the 2015 North American Land Change Monitoring System 30 m × 30 m land‐cover dataset, produced from Landsat satellite imagery (Commission for Environmental Cooperation 2020).

SnowModel is founded on realistic representations of the physics controlling snow evolution (Liston et al. 2020). For each spatiotemporal scale, we modeled air temperature, wind speed, amount of rainfall, amount of snowfall, snow water equivalent (SWE) depth, SWE melt, snow density, snow depth, snow‐surface temperature, temperature of each snowpack layer, and amount of runoff from the base of the snowpack. To ensure the highest quality estimated snow conditions, we also assimilated in situ SWE and snow depth observations from 77 Natural Resources Conservation Service snow telemetry (SNOTEL) and snow course sites within our study domain (Liston and Hiemstra 2008; Serreze et al. 1999; Stuefer, Kane, and Liston 2013) (Figure 2).

SnowModel can create nuanced, project‐specific snow data products required for specific applications (Boelman et al. 2019; Glass et al. 2021; Reinking et al. 2022). In situ snow crust data were not readily available for our study period and domain. Therefore, we used knowledge of the physical processes underlying snow crust formation to develop a comprehensive snow crust evolution submodel (*sensu* Reinking et al. 2022). Specifically, using the aforementioned SnowModel output variables, we implemented a physics‐based model that simulated the spatiotemporal evolution of snow crusts associated with the three main phenomena that produce them: melt‐freeze, rain‐on‐snow, and blowing and drifting snow associated with strong wind events (Fierz et al. 2009; Woo and Heron 1981).

#### 2.3.1 Melt‐Freeze Ice Crust

Melt‐freeze crusts form when snow melts and then refreezes either at the surface or within the snowpack. We identified ice crusts that formed during a melt‐freeze cycle by calculating, at each model time step, how much snowmelt occurred. Then, we assumed any meltwater produced would freeze if either the surface or any snowpack layer temperature was below a defined snow temperature threshold within the subsequent 12 h period; this 12 h period accounted for the role of day–night snow‐temperature variations in ice crust formation. We assumed that if snowmelt water ran out of the bottom of the snowpack, a melt‐freeze layer did not form.

First, we calculated the formation and severity of each melt crust (*crust event severity*). A melt‐crust event severity, *M*_e_ (m), at each model time step, *t* (dimensionless), was defined to equal the snowmelt produced by SnowModel during that time step, *M* (m), for any case where the snowpack had a depth ≥ 5.0 cm (i.e., the presumed depth of snow necessary to create subnivean habitat for small mammals such as *T. talpoides* and *Microtus* species) and snow‐layer temperature, *T*_s_ (°C), below the snow temperature threshold, *T*_s_threshold_ (= −1.0°C).

(1) $$M_{e}\left(t\right)=\left\{\begin{array}{cc} M\left(t\right); & T_{s}\left(t\right)<{{T_{s}}_{\_}}_{\text{threshold}}\\ 0.0\;\;; & T_{s}\left(t\right)\geq {{T_{s}}_{\_}}_{\text{threshold}} \end{array}\right.$$

The snow layer temperature was defined to be the lowest snow temperature in any of SnowModel's snowpack layers at the current time step, *t*. The threshold temperature identifies whether the snowpack is cold enough to freeze liquid water (i.e., the snowpack requires a cold content, so this threshold must be below 0.0°C). A threshold value of −1.0°C was chosen to be consistent with general snowpack temperature simulation uncertainty following Liston et al. (2020).

In our modeling system, once a crust is formed, it persists, even as new snow falls and buries it below the snow surface. Thus, the crust sub‐model assumed that crust‐formation events were cumulative throughout the winter (*cumulative crust severity*). This cumulative crust measure was assumed to persist in the snowpack until the entire snowpack was isothermal (0.0°C throughout), melting, and water was running out the bottom of the snowpack (typically in the spring or, in low elevations, periodically throughout the winter). This condition occurs when SnowModel's snowmelt runoff variable, *R* (*m*), is non‐zero. Once runoff occurred, we reset the cumulative crust severity to zero. Thus, the cumulative melt‐crust severity, *M*_*c*_ (m), is given by

(2) $$M_{c}\left(t\right)=\sum_{t=1}^{t=t_{\max}}\left\{\begin{array}{l} M_{e}\left(t\right)+M_{c}\left(t-1\right);\;R\left(t\right)=0.0\\ 0.0\;\;\;;\;R\left(t\right)>0.0 \end{array}\right.$$

where *t*_max_ is the number of time steps in an annual SnowModel simulation.

The melt‐crust sub‐model also counted the number of consecutive time steps, *M*_p_ (days), that had a non‐zero melt crust (*crust persistence*). This is given by

(3) $$M_{p}\left(t\right)=\sum_{t=1}^{t=t_{\max}}\left\{\begin{array}{l} M_{p}\left(t-1\right)+1.0\;;\;M_{c}\left(t\right)>0.0\\ 0.0\;\;\;\;\;\;;\;M_{c}\left(t\right)=0.0 \end{array}\right.$$

For sub‐daily SnowModel outputs, *M*_p_ is converted to daily values by multiplying it by Δ*t_*out/24, where Δ*t_*out is the SnowModel output time increment in hours.

#### 2.3.2 Rain‐on‐Snow Ice Crust

Rain‐on‐snow crusts form when liquid precipitation (rain) falls on a cold (below freezing) snow surface or on a snowpack with cold internal snow layers, and the rain freezes into an ice layer. We identified events during which rain (≥ 1.0 mm) fell on a cold snowpack (i.e., a snowpack with a depth ≥ 5.0 cm and a snowpack layer with a temperature < *T*_s_threshold_), and the rain froze in the surface layer or layers below, and did not produce runoff.

Thus, the rain‐on‐snow (ROS) ice crust was simulated in a similar fashion to the melt‐freeze ice crust formulation. The ROS‐crust event quantity, ROS_e_ (*m*), at model time step, *t*, was defined to equal the rain produced by SnowModel during that time step, *P* (m), for any case where the snowpack had a depth ≥ 5.0 cm and snow‐layer temperature, *T*_s_ (°C), below the snow temperature threshold, *T*_s_threshold_. The equation describing a ROS crust‐formation event is given by Equation (1), where *M*_e_ is replaced by ROS_e_, and *M* is replaced by *P*. A non‐zero ROS‐crust event indicates that a ROS crust of that magnitude formed at that time step. The ROS‐crust sub‐model assumes that crust‐formation events are cumulative throughout the winter. Thus, the cumulative ROS‐crust severity, ROS_c_ (m), is given by Equation (2), where *M* is replaced by ROS. The ROS‐crust sub‐model also counts the number of consecutive time steps, ROS_p_ (days), that had a non‐zero ROS crust. This is given by Equation (3), where *M* is replaced by ROS.

#### 2.3.3 Ice Crust

Because melt‐freeze and rain‐on‐snow crust indices were comparable in scale and formation process, for the purposes of our analyses, we merged the melt‐crust and ROS‐crust event variables described above into a single liquid‐freeze (ice) crust event variable, *L*_e_ (m). The melt‐crust and ROS‐crust variables were combined in the following way,

(4) $$L_{e}\left(t\right)=M_{e}\left(t\right)+{\mathrm{ROS}}_{e}\left(t\right)$$

Then the cumulative liquid‐freeze crust severity, *L*_c_, and crust persistence, *L*_p_, were created following Equations (2) and (3), respectively, where *M* is replaced by *L*. In addition, because the ice crust event and cumulative severity variables yielded small numbers (with units of m, consistent with the SnowModel snowmelt and rainfall outputs) that were hard to visualize (on the order of 0.001 m), these variables were multiplied by 1000 to create a relative index where 1.0 represents an ice crust thickness of 1.0 mm. Thus, indices of melt‐freeze and ROS crust severity were based on the amount of liquid water (snowmelt or rainfall, in millimeters) that reached a < *T*_s_threshold_ snowpack layer. For example, an ice crust severity index of 10.0 meant 10.0 mm of liquid water had frozen.

#### 2.3.4 Wind Crust

Wind crust formation occurs when high‐speed winds (e.g., winds generally greater than 5 to 7 m s^−1^; Li and Pomeroy 1997; Liston and Sturm 1998) interact with falling or blowing snow. Such events result in the mechanical breakage or tumbling of snow into small, sometimes sharp‐edged crystals that can form strong, grain‐to‐grain bonds, through a process called sintering (Blackford 2007; Colbeck 1997), once they stop moving. To model wind crust events, we calculated the relative increase in snow density based on the wind speed 2 m above the land surface and the amount of blowing snow, following the wind‐related snow density evolution formulation in Liston et al. (2007, 2020).

The wind crust has units of snow density (kg m^−3^), and wind crusts are only produced for snowpacks > 5.0 cm deep. Creation of the wind crust variable first requires converting the SnowModel wind field, *U*_obs_ (m s^−1^), at height, *z*_obs_ (m), to a common height (*z*_2m_ = 2.0 m). This is done using

(5) $$U_{2m}=U_{\mathrm{obs}}\frac{\text{ln}\left(\frac{Z_{2m}}{Z_{0}}\right)}{\text{ln}\left(\frac{Z_{\mathrm{obs}}}{Z_{0}}\right)}$$

where *U*_2m_ (m s^−1^) is the wind speed at 2 m height, and *z*_0_ (m) is the snow surface roughness (assumed *t*_o_ = 0.001 m). The wind crust can then form under two different conditions: new snowfall under blowing‐snow conditions, *W*_bs1_ (kg m^−3^), and snow blowing without new snow falling, *W*_bs2_ (kg m^−3^).

During snowfall, a wind crust forms in the newly created top snow layer through the influence of blowing and drifting snow. For wind speeds ≥ 5 m s^−1^, the new‐snow wind crust contribution from blowing‐snow, *W*_bs1_ (kg m^−3^), is given by

(6) $$W_{\mathrm{bs}1}=C_{1}+C_{2}\left\{1.0-\exp\left[-C_{3}\left(U_{2m}-5.0\right)\right]\right\}$$

where *C*_1_, *C*_2_, and *C*_3_ are constants set equal to 25.0 kg m^−3^, 250.0 kg m^−3^, and 0.2 s m^−1^, respectively; *C*_1_ defines the density offset for a 5.0 m s^−1^ wind (i.e., the wind crust density for a 5.0 m s^−1^ wind speed), *C*_2_ defines the maximum density increase due to wind, and *C*_3_ controls the progression from low to high wind speeds (Liston et al. 2007, 2020). The wind crust index represents the blowing‐snow‐related contribution to snow density evolution. Numerous other factors and processes also modify the snow density, including time, snow temperature, rain on snow, snowmelt, the weight of the overlying snow, and temperature and vapor pressure gradients within the snowpack (e.g., Liston et al. 2020). Hence, the wind crust index density values and *C*_2_ are typically less than the snowpack snow density values.

If the new‐snow wind crust, at the current SnowModel time step, *t*, has a higher value than the final wind crust value at the previous time step, *t*−1, then the wind crust metric is updated following,

(7) $$W_{\mathrm{bs}2}\left(t\right)=W_{\mathrm{bs}1}\left(t\right);\;\;\;\text{if}\;\;\;W_{\mathrm{bs}1}\left(t\right)>W_{\mathrm{bs}2}\left(t-1\right)$$

Wind speed at the current time step also contributes to wind crust formation during periods of no precipitation, *W*_bs2_ (kg m^−3^). In this case, the top snow layer wind crust evolves similarly to the snow density evolution defined by Andersen and Gill (1982), but with a wind‐speed contribution, *U*. This temporal change in snow crust from blowing snow is updated using

(8) $$\frac{{\mathrm{dW}}_{\mathrm{bs}2}}{\mathrm{dt}}=m_{1}\;m_{2}\;U\;W_{\mathrm{bs}2}\;\exp\left(-m_{3}\;W_{\mathrm{bs}2}\right)$$

which is discretized as follows

(9) $$W_{\mathrm{bs}2}\left(t\right)=W_{\mathrm{bs}2}\left(t-1\right)+m_{1}\;m_{2}\;\mathrm{\Delta t}\;U\;W_{\mathrm{bs}2}\left(t-1\right)\exp\left(-m_{3}\;W_{\mathrm{bs}2}\left(t-1\right)\right)$$

where Δ*t* is the model time step in seconds, *m*_1_ = 0.0005 is a non‐dimensional constant that controls the simulated snow crust change rate, and *m*_2_ = 0.0013 and *m*_3_ = 0.021 following Andersen and Gill (1982).

For wind speeds ≥ 5 m s^−1^, *U* is given by

(10) $$U=E_{1}+E_{2}\left\{1.0-\exp\left[-E_{3}\left(U_{2m}-5.0\right)\right]\right\}$$

with *E*_1_, *E*_2_, and *E*_3_ defined to be 5.0 m s^−1^, 15.0 m s^−1^, and 0.2 s m^−1^, respectively; *E*_1_ defines the *U* offset for a 5.0 m s^−1^ wind, *E*_2_ defines the maximum *U* increase due to wind, and *E*_3_ controls the progression of *U* from low to high wind speeds (Liston et al. 2007, 2020). For wind speeds < 5 m s^−1^, *U* is defined to equal 0.0 m s^−1^. This approach limits the crust value increase resulting from wind transport to winds capable of moving snow (assumed to be winds ≥ 5 m s^−1^).

The above wind crust formulation is similar to the cumulative melt crust severity variable; it continues evolving in time and is set to zero when snowmelt water is running out of the snowpack as runoff. Therefore, we define the cumulative wind crust severity, *W*_c_ (kg m^−3^), to be,

(11) $$W_{c}\left(t\right)=\sum_{t=1}^{t=t_{\max}}\left\{\begin{array}{l} W_{\mathrm{bs}2}\left(t\right);\;R\left(t\right)=0.0\\ 0.0\;\;\;\;;\;R\left(t\right)>0.0 \end{array}\right.$$

This can be used to extract the time evolution of wind crust events, *W*_e_ (kg m^−3^), using the formula

(12) $$W_{e}\left(t\right)=\left\{\begin{array}{l} W_{c}\left(t\right)-W_{c}\left(t-1\right);\;W_{c}\left(t\right)-W_{c}\left(t-1\right)>0.0\\ 0.0\;\;\;\;\;\;\;;\;W_{c}\left(t\right)-W_{c}\left(t-1\right)\leq 0.0 \end{array}\right.$$

The wind‐crust sub‐model also counts the number of consecutive time steps, *W*_p_ (days), that had a non‐zero wind crust. This is given by Equation (3), where *M* is replaced by *W*.

The severity indices for ice crusts versus wind crusts are not directly comparable due to the inherent differences in how these crusts form in the natural system. For this reason, we did not combine the ice and wind crusts into a single, all‐encompassing crust index. Note that our snow crust severity estimates do not equate to specific measurements of snow crust hardness, strength, or density. Rather, our ice and wind crust indices represent the current state‐of‐the‐art understanding of the physical mechanisms that control snow crust formation and evolution (Serreze et al. 2021).

### 2.4 Habitat Selection

We used integrated step‐selection analysis (iSSA) (Avgar et al. 2016) to evaluate how snow conditions (Table 1) influenced proximate habitat selection by Great Gray Owls during winter. In contrast to traditional resource selection functions (RSFs), which compare use versus availability at a designated spatial scale (e.g., the home range), iSSA evaluates habitat selection at the scale of the movement step, with each step consisting of both a starting and ending location between a set time interval. This analysis assumes that, for each observed step, alternate available steps exist that the animal could have selected (Avgar et al. 2016; Thurfjell, Ciuti, and Boyce 2014). Integrated step‐selection analysis accounts for the autocorrelation inherent in movement data and, therefore, is a suitable modeling approach to evaluate fine‐scale habitat selection decisions (Avgar et al. 2016).

**TABLE 1:** Snow characteristics used to assess winter step‐selection and time‐to‐departure for long‐distance movements by adult Great Gray Owls (*n* = 42) during 2017–2022 in the Greater Yellowstone Ecosystem, Wyoming, USA.

Snow characteristics
Snow depth
Wind crust event severity
Cumulative wind crust severity
Wind crust persistence
Ice crust event severity
Cumulative ice crust severity
Ice crust persistence

*Note:* Snow conditions were estimated using SnowModel and were generated at a 3 h time step and a 30 m × 30 m resolution for fine‐scale step‐selection analysis and a 500 m × 500 m resolution for analysis of broader‐scale, long‐distance movements. Ice crusts were quantified by combining snow crusts formed by rain‐on‐snow and melt‐freeze events.

We subsampled Great Gray Owl GPS location data to include the core snow period (defined as the average dates of the longest period during which snow cover was present; 15 September to 15 April) and one location per day (to standardize sample rates across tagged owls), such that successive locations were 24 h apart. We generated five corresponding, random, available steps for each observed step (Northrup et al. 2013; Thurfjell, Ciuti, and Boyce 2014), using a Weibull distribution (Forester, Im, and Rathouz 2009) and von Mises distribution (Marsh and Jones 1988) for step length and turning angle, respectively. At the endpoint of each step, we extracted values of snow depth; wind crust metrics (event severity, cumulative severity, and persistence); and ice crust metrics (event severity, cumulative severity, and persistence). We centered and scaled environmental covariates to allow for the comparison of effect sizes, or relative selection strength (RSS), between variables (Avgar et al. 2017).

We parameterized models using conditional logistic regression to analyze the selection of step endpoints, presenting results as the natural logarithm of RSS (log‐RSS). We assigned a unique cluster to each individual by year. We used Pearson's correlation coefficients to test for collinearity between environmental covariates and considered |*r*| ≥ 0.7 to be significantly correlated. If two variables were correlated, we did not include both variables in the same model. We parameterized a null, base model that only contained step distance and a full model that contained step distance, snow depth, wind crust event severity, cumulative wind crust severity, ice crust event severity, cumulative ice crust severity, and ice crust persistence. We added additional, individual variables from the full model to the null model using a forward step‐wise approach based on their significance using Akaike's Information Criteria (AIC). We calculated variance inflation factors (VIFs) for all variables within a given model to evaluate collinearity between variables and ensured all VIFs were < 2 (O'Brien 2007). To evaluate the relative empirical support for each model, we used an information‐theoretical approach based on quasi‐likelihood under independence criterion (QIC), which considers non‐independence within a given cluster, and we identified the top model as that with the lowest QIC value. We calculated robust standard errors and 95% confidence intervals using generalized estimating equations to account for temporal autocorrelation between each individual's movements (Craiu, Duchesne, and Fortin 2008). We conducted analyses in mathematical software R (Version 3.4.2; R Core Team 2021) and prepared data for the iSSA using the MoveTools package (Version 0.0.0.9001; Merkle et al. 2022).

### 2.5 Long‐Distance Movements

To evaluate whether snow conditions (Table 1) precipitated longer‐distance movements (including nomadic movements), we identified long‐distance movement events away from home ranges by Great Gray Owls during the core snow period (15 September to 15 April). We used net squared displacement (NSD) analysis (Bunnefeld et al. 2011), which characterizes movement trajectories by calculating the squared Euclidean distance between a starting location and subsequent locations. We also visually inspected GPS location data to confirm that long‐distance movement events met specific criteria. We identified a long‐distance movement event as a significant movement away from a discrete breeding or winter range. A significant movement was defined as a movement of 8 km or farther, based on twice the diameter of the mean overall winter range area (19.5 km^2^), based on 99% Kernel Density Estimates (KDE) (Gura 2023 ).

We defined breeding home ranges based on 95% KDE using owl locations from 1 April to 30 September (the breeding season) (Gura 2023). A discrete winter home range was defined as an area < 3.7 km in diameter (based on a mean winter home range size of 13.4 km^2^ for 95% KDE; Gura 2023) in which an owl settled for a minimum of 1 week (between 1 October and 31 March). Although we included significant movements away from breeding ranges and significant movements from discrete winter ranges to another wintering area, we excluded movements in which owls departed discrete winter ranges to *return* to their breeding range, because we could not ascertain whether factors other than snow conditions (e.g., acquisition and defense of a territory and mate; Currie, Thompson, and Burke 2000; Forstmeier 2002) influenced their decisions. We excluded movement sequences with > 5 days of missing location data or < 6 months of GPS location data total.

For each long‐distance movement event, we identified the onset of risk as the point when the owl first settled in either a discrete breeding or winter range (settlement decision), and included all locations in which the owl remained settled (settlement) up until the point when the owl departed the settled range to undertake a long‐distance movement (departure event). For all locations beginning with the settlement decision through the departure event, we extracted values of snow depth and crust event severity, cumulative crust severity, and number of consecutive days each individual owl experienced crust conditions (crust persistence), for both wind and ice crusts. For each location, we also extracted the summed severity of wind crust events and summed severity of ice crust events during the prior 7 days, to test for a lag effect of crust events on departure. We used Cox proportional hazards (CPH) analysis (a hazard model that analyzes time‐to‐event data) (Andersen and Gill 1982; Cox 1972) to evaluate whether snow conditions influenced the probability of departure. We calculated the baseline hazard function of time‐to‐departure (Dossman et al. 2016) using hazard regression to determine the probability of a long‐distance movement occurring at a given point in time, using time‐varying snow conditions as explanatory variables.

We stratified the data by assigning a unique cluster to each individual‐by‐year to account for any additional variation in the probability of departure associated with the individual and annual variation. We tested for correlation among predictor covariates using Pearson's correlation (with |*r*| ≥ 0.7 considered significantly correlated) and confirmed that VIFs were < 2. We used a bootstrap, stepwise model‐selection approach based on Akaike's Information Criteria value, adjusted for small sample size (AICc) (Austin and Tu 2004; Hosmer, Lemeshow, and May 2008) using the Program R package “bootStepAIC” (Rizopoulos 2022). Here, we used a bootstrap procedure in which a sub‐sample of the dataset was randomly generated, the full model (containing the variables within Table 1) was re‐fit using this sub‐sampled dataset, and a backward, step‐wise model selection procedure was conducted. This entire process was replicated 100 times to identify the top, most parsimonious model based on the lowest AICc value. We confirmed that model covariates exhibited a linear relationship with the hazard ratio using a generalized additive model to evaluate the effective degrees of freedom (EDF) of the covariates, and we ensured that covariates and the top model met the assumption of proportionality using Schoenfeld residuals (Grambsch and Therneau 1994; Hosmer, Lemeshow, and May 2008; Schoenfeld 1982). We conducted CPH analysis in R using the Survival package (Version 3.5.5; Therneau 2023).

## 3 Results

### 3.1 Owl Data

We captured and deployed GPS transmitters on 42 adult Great Gray Owls from November 15, 2017 to September 1, 2021. We collected 135,087 total GPS locations for 22 male and 20 female owls between November 15, 2017 and September 31, 2022. These data included locations for 69 individuals‐by‐year and 21,113 locations during the core snow period.

### 3.2 Snow Data

**FIGURE 3:**
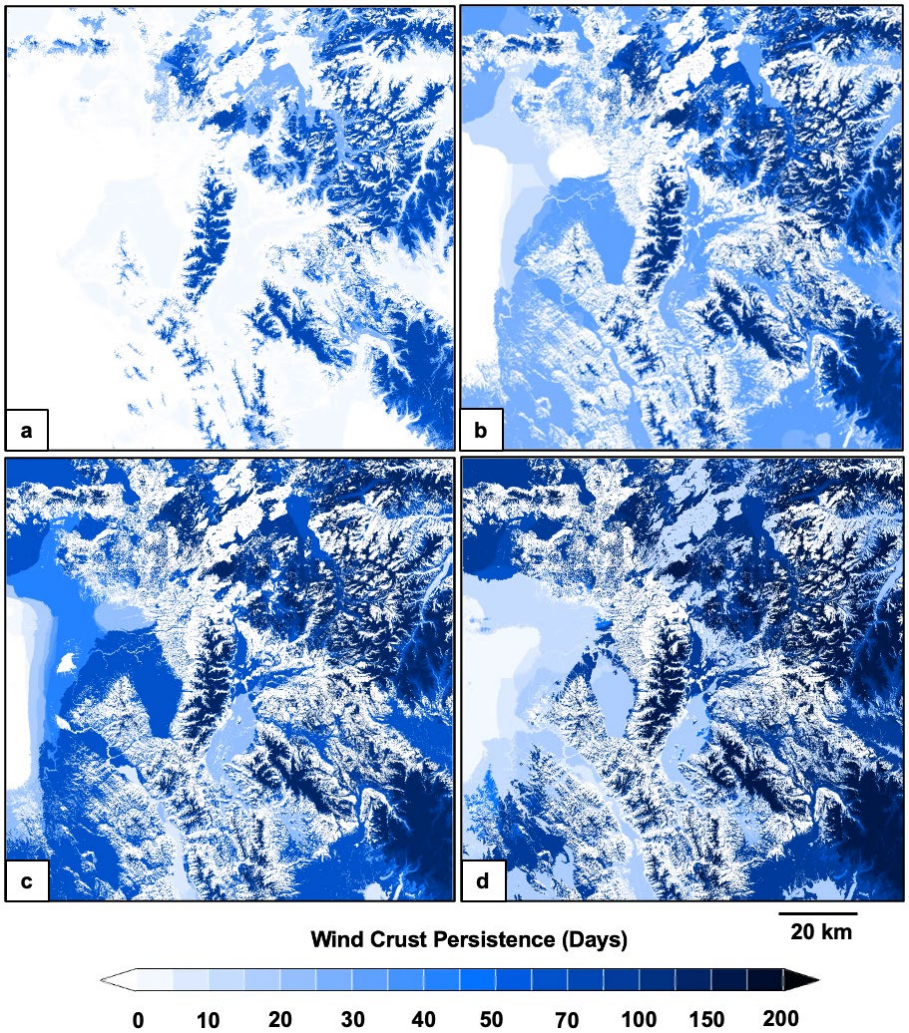
Spatiotemporal evolution of wind crust persistence across the Greater Yellowstone Ecosystem on December 1, 2017 (a), January 1, 2018 (b), February 1, 2018 (c), and March 1, 2018 (d). Snow crust persistence was derived from estimated indices of snow crust severity, modeled at a 3‐h time step and 30 m × 30 m spatial resolution using SnowModel.

**FIGURE 4:**
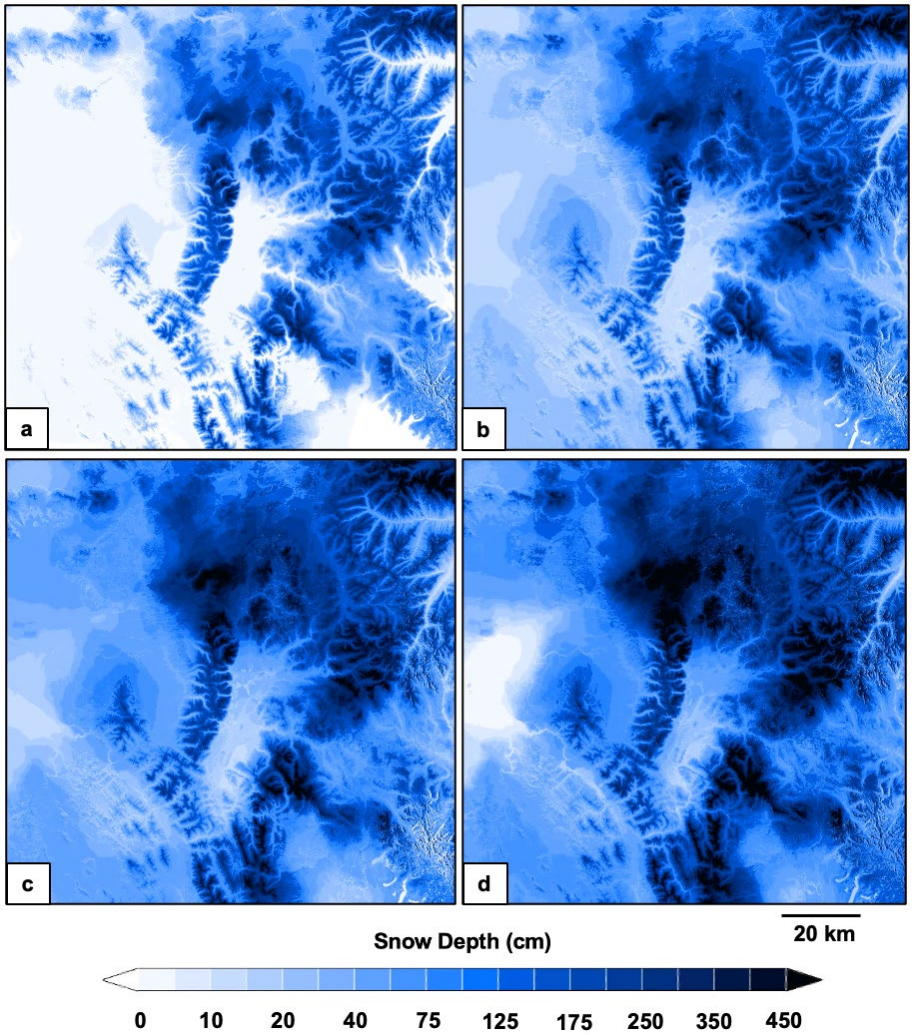
Spatiotemporal evolution of snow depth across the Greater Yellowstone Ecosystem on December 1, 2017 (a), January 1, 2018 (b), February 1, 2018 (c), and March 1, 2018 (d). Snow depth was modeled at a 3‐h time step and 30 m × 30 m spatial resolution using SnowModel.

**FIGURE 5:**
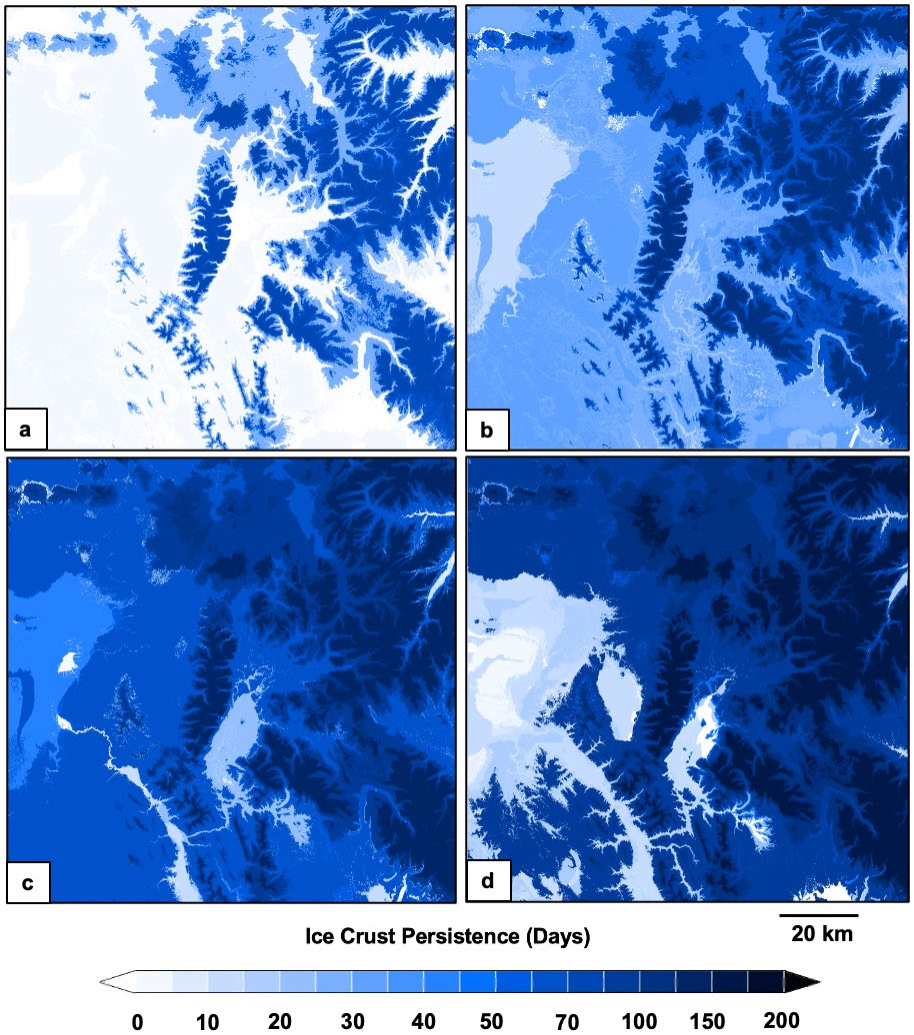
Spatiotemporal evolution of ice crust persistence across the Greater Yellowstone Ecosystem on December 1, 2017 (a), January 1, 2018 (b), February 1, 2018 (c), and March 1, 2018 (d). Snow crust persistence was derived from estimated indices of snow crust severity, modeled at a 3‐h time step and 30 m × 30 m spatial resolution using SnowModel.

**FIGURE 6:**
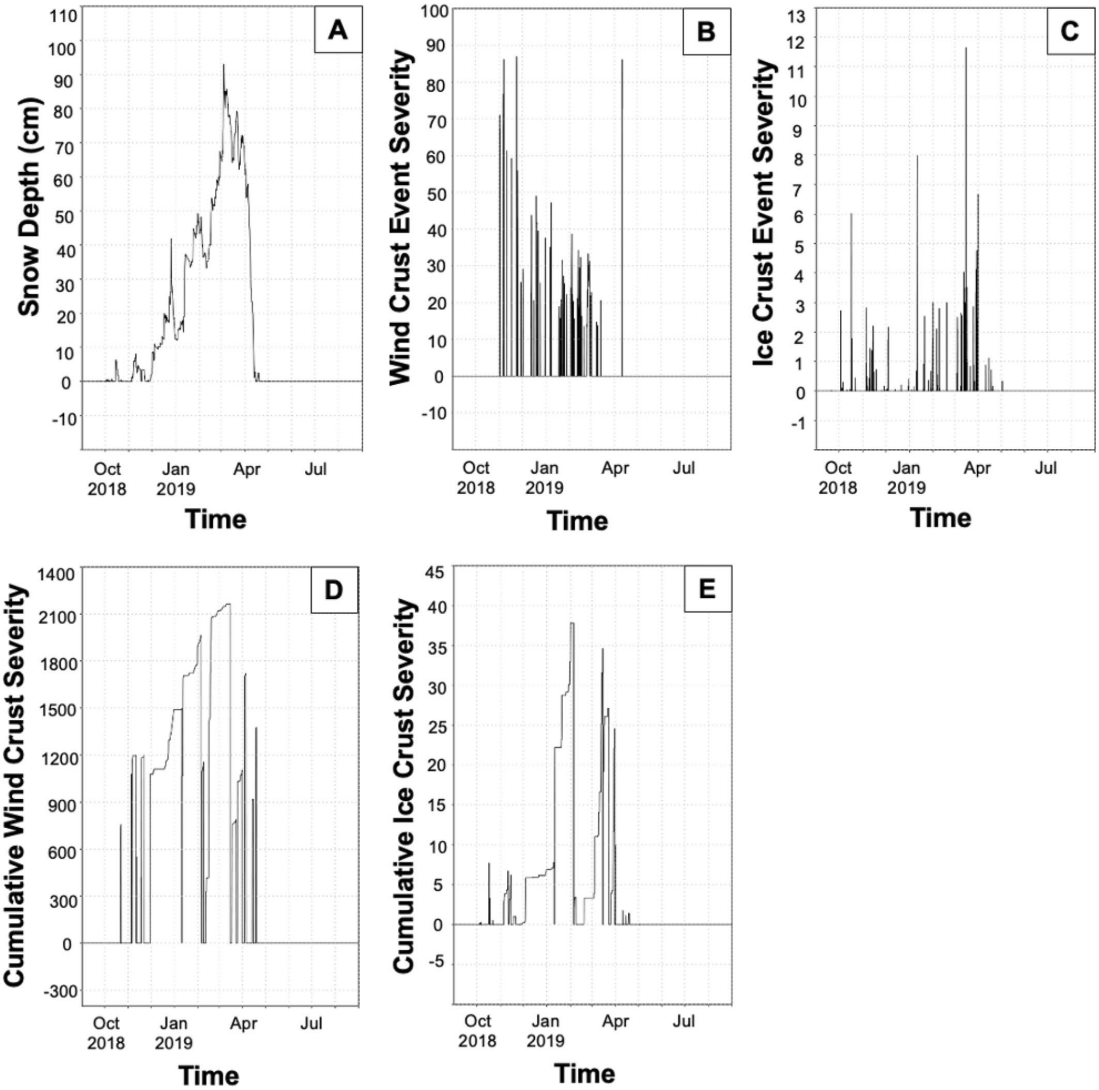
Example time series of snow depths (A), wind crust events (B), ice crust events (C), cumulative wind crust severity (D), and cumulative ice crust severity (E) at one point in space in the Greater Yellowstone Ecosystem from September 1, 2018 to August 31, 2019. Snow conditions were modeled at a 3‐h time step and 30 m × 30 m spatial resolution using SnowModel.

The spatiotemporal distribution of modeled snow characteristics varied across the study domain. Wind crusts exhibited high spatial heterogeneity (Figure 3a–d), snow depths were less spatially variable (Figure 4a–d), and ice crusts were relatively homogenously distributed across broad areas (Figure 5a–d). Snow depths increased steadily from early winter until mid‐spring (Figure 6A). Crust‐producing wind events occurred throughout the core snow periods (Figure 6B). Ice crust events occurred primarily during the early winter and early spring periods when temperatures tended to be relatively higher (resulting in increased rain‐on‐snow and melt‐freeze events), although ice crusts occasionally occurred mid‐winter (Figure 6C). Both wind and ice crusts persisted for extensive periods of time, although periodic mid‐winter runoff events occurred that dissolved wind and ice crusts (Figure 6D,E).

### 3.3 Habitat Selection

**FIGURE 7:**
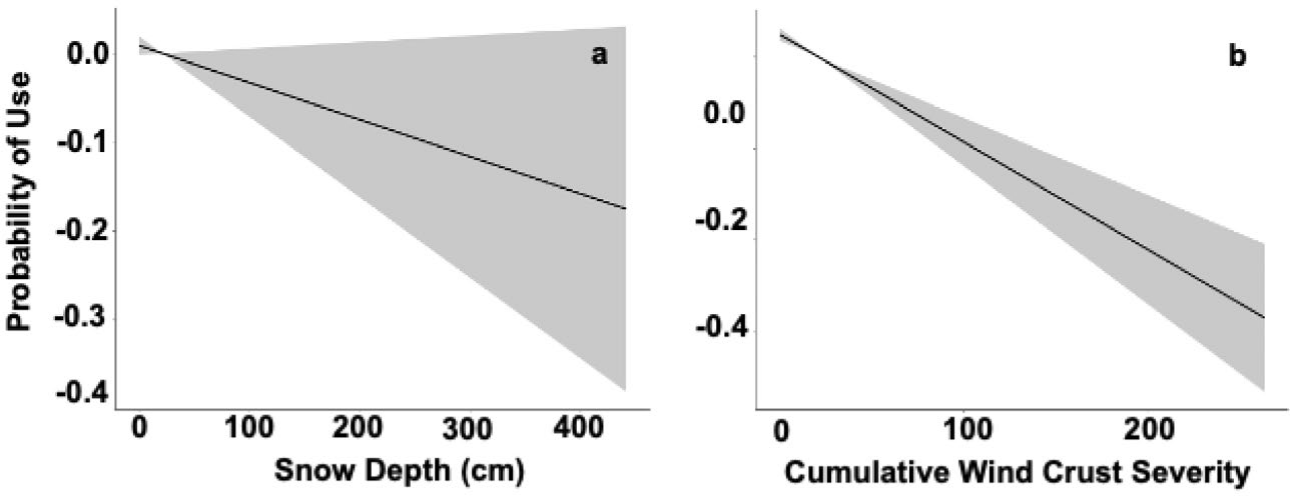
Probability of use of varying snow depths (a) and cumulative wind crust severity (b) by Great Gray Owls (*n* = 42) in the Greater Yellowstone Ecosystem during the winters of 2017–2022. Estimates are based on integrated step selection analysis (iSSA) at the daily step level. Gray shading indicates 95% confidence intervals, and probabilities are based on the mean coefficients of iSSA.

We evaluated 2,946 daily steps of Great Gray Owls during winter, which we compared to 14,700 available step locations. The average daily step‐length was 1,321 m (± 2,001 m; range: 2–23,607 m). Wind crust conditions and snow depth best explained fine‐scale habitat choices (Table 2, Table A1). Owls strongly avoided areas with increased cumulative wind crust severity and snow depth (Table 2, Figure 7). In contrast, proximate habitat choices did not vary with ice crust conditions (event severity, cumulative severity, or persistence).

**TABLE 2:** Top model summarizing environmental covariates influencing proximate selection of environmental variables at the daily step level by adult Great Gray Owls (*n* = 42) in the Greater Yellowstone Ecosystem during the winters of 2017–2022.

Explanatory variable	Estimate	*p*	95% CI	
Snow depth	−0.008	< 0.001	−0.011	−0.005
Cumulative wind crust severity	−0.004	< 0.001	−0.005	−0.003

*Note:* Results are based on population‐level averages of integrated step selection analysis coefficient estimates for probability of use of snow depth and cumulative wind crust severity at the endpoint of daily steps. Here, we report the output coefficient (estimate), *p*‐value, and 95% confidence intervals (CI) for covariates in the top model. The degrees of freedom = 3 (including step‐length), *n*_events_936, and the Wald Test = 148.2 (*p* = < 0.001).

### 3.4 Long‐Distance Movements

We analyzed the long‐distance movement behavior of 36 owls following the sub‐sampling of data. Five owls did not depart during the winter and remained in their breeding range throughout the entire annual cycle. We identified 139 long‐distance movement events for the remaining 31 owls (15 males and 16 females) across years (109 individuals‐by‐year). The farthest observed movement was 112 km. Long‐distance movements occurred as early as 28 September and as late as 10 April. The mean date of first departure was 5 November (± 32 days), and the average day of first departure was 25 October (± 22 days) and 14 November (± 36 days) for female and male owls, respectively. The mean number of departure events per individual‐by‐year was 3.1 (± 2; range: 1–8). Most owls exhibiting long‐distance and nomadic movements repeatedly returned to and left breeding ranges over the course of the winter. All environmental covariates included in the global CPH model met the assumptions of linearity (EDF = 1.14–1.27) and proportionality (*p* = 0.98–1.00).

Snow depth and ice crust conditions best explained the initiation of long‐distance movements of owls during winter (Table A2, Table 3). The probability of long‐distance movements increased with the depth of snow at the time of departure (HR = 1.010, CI = 1.007–1.014, *P* = < 0.001) and the severity of ice crust events during the previous week (HR = 1.060, CI = 0.998–1.125, *p* = 0.059) (Table 3). The probability that an owl would initiate a long‐distance movement increased by 1% for every centimeter increase in snow depth, and by 6% for every additional millimeter of water converted into an ice crust within the week prior. Wind crust event severity during the prior week was included in the top model, although there was no evidence that it determined the time of departure (HR = 1.707, CI = 0.882–3.303, *p* = 0.113) (Table 3). Likewise, the severity of wind crust and ice crust events at the time of departure both were included in competing models of time‐to‐departure (ΔAICc < 2) (Table A2) but did not influence the probability of a long‐distance movement (HR = 0.244, CI = 0.004–16.290, *p* = 0.510; HR = 1.190, CI = 0.789–1.796, *p* = 0.407).

**TABLE 3:** Environmental covariates influencing the probability of winter long‐distance movements by adult Great Gray Owls (*n* = 42) in the Greater Yellowstone Ecosystem during the winters of 2017–2022.

Explanatory variable	Estimate	HR	SE	*p*	95% CI	
Ice crust event severity (prior week)	0.058	1.060	0.031	0.059	0.998	1.125
Snow depth	0.010	1.011	0.002	< 0.001	1.007	1.014
Wind crust event severity (prior week)	0.535	1.707	0.337	0.113	0.882	3.303

*Note:* Results reflect the most parsimonious model based on AICc value for Cox proportional hazards analysis evaluating time‐to‐departure. Here, we report the output coefficient (estimate), hazard ratio (HR), standard error (SE), *p*‐value, and 95% confidence intervals (CI) for covariates in the top model. The degrees of freedom = 3, *n*_events_ = 139, and the Wald Test = 37.31 (*p* = < 0.001). “Prior Week” ice crust and wind crust event severities refer to the summed crust events that occurred within the 7 days prior to departure.

## 4 Discussion

Determining how organisms cope with unpredictable and extreme conditions that affect fitness outcomes is key to understanding species distribution and persistence in a rapidly changing world. The degree and scale of limiting conditions likely comprise primary factors modulating responses but to a largely unknown extent. Using the Great Gray Owl in the GYE as a model system, and a state‐of‐the‐art environmental modeling approach, we evaluated whether the severity and spatiotemporal extent of limiting conditions regulated movement strategies. Owls proximately avoided deeper and wind‐compacted snow via shifts in immediate, local habitat use. Individuals were more likely to undertake long‐distance movements in response to deeper snow and more severe ice crust events that impacted broader regions. Our results showcase facultative movement behavior to avoid snow conditions that restrict food access.

We documented a facultative migrant using both proximate shifts in habitat selection and broad‐scale, long‐distance and nomadic movements to mediate limiting conditions that vary in terms of spatiotemporal extent and severity. Snow depth, wind crusts, and ice crusts manifest via unique mechanisms that result in different spatiotemporal dynamics. For example, areas susceptible to wind crusts include high elevations, windward aspects, ridges, and open habitats (Liston et al. 2007), and wind crusts were relatively heterogeneously distributed within the GYE because of the region's variation in elevation, topography, and land‐cover type. As a result, owls were able to locate and utilize local refuges from wind‐compacted snow on a daily basis. In contrast, the rain‐on‐snow and melt‐freeze events that create ice crusts largely are influenced by broad‐scale temperature and precipitation regimes (Rennert et al. 2009). Thus, ice crusts generally occurred across large swaths of the GYE. As a result, refuges from ice crusts rarely existed locally, which likely necessitated more extensive, long‐distance movements by owls. The general hypothesis that broad‐scale environmental structuring determines migratory movements (Bastille‐Rousseau et al. 2017; Mueller et al. 2011; Teitelbaum et al. 2015) therefore likely applies to facultative migrants.

Our results demonstrate that movement behavior is context‐dependent, with proximate habitat selection and longer‐distance movements both serving as viable responses to limiting conditions. Owls utilized both local shifts in habitat selection and long‐distance movements in response to deeper snow that was relatively moderately‐distributed. Although owls capitalized on local refugia in the form of shallower snow, such refugia may not be sufficient to offset limiting conditions, ultimately requiring longer‐distance movements to areas of suitable habitat. Indeed, in this system, what constituted suitable habitat was highly dynamic in space and time, which influenced the distribution of an apex predator and likely other organisms across trophic levels.

The specific external (e.g., precipitation and impenetrable snow) and/or internal (e.g., body condition and stress hormones) cues that precipitate facultative movement responses warrant further examination. The majority of research on predictive cues for migratory behavior has been conducted on obligate migrants within temperature latitudes, where environmental factors (e.g., photoperiod, temperature, precipitation, barometric pressure, or wind direction and speed) can combine with intrinsic factors such as physiological state and energy reserves to influence movement decisions (Newton 2008; Cornelius et al. 2013). In addition to extreme changes in environmental conditions, corticosterone (a glucocorticoid linked to stress response and metabolism) is thought the be an important determinant of avian facultative movements (Breuner, Wingfield, and Hahn 1998), such as the altitudinal migrations undertaken by White‐Crowned Sparrows ( *Zonotrichia leucophrys* ) in response to spring snowstorms (Breuner et al. 2003). Owls in our study did not depart immediately in response to crust events; rather, we observed a lagged response in which crust events during the prior week best explained the timing of long‐distance movements. This delay in response suggests that rather than the crust event (e.g., a rain‐on‐snow event) serving as a cue to leave (resulting in a predictive evasive movement), owls likely initiated a reactive evasive movement because of an inability to capture sufficient food resources (Watts and Cornelius 2024).

How snow conditions that limit access to food interact with prey abundance and distribution to influence predator movements also warrants further investigation. During the breeding‐season in the GYE, Great Gray Owls primarily preyed upon pocket gophers (Franklin 1988; Gura 2023), which exhibited relatively stable population dynamics from year‐to‐year (Gura 2023). However, during winter, owls may be more reliant on voles (*Microtus* spp.), which exhibit relatively cyclical population dynamics (Fauteux et al. 2015; Lambin, Petty, and MacKinnon 2000). In general, information on the winter distribution and abundance of small mammals in the GYE is lacking. Across more northern‐latitude boreal forests, Great Gray Owl irruptions are thought to occur in response to low vole abundance (Bull and Henjum 1990; Graves et al. 2012; Hipkiss, Stefansson, and Hörnfeldt 2008). Likewise, how breeding status influences facultative movement decisions remains an important avenue of research. In our study, several individuals remained on breeding ranges year‐round and most owls that left repeatedly returned to breeding ranges over the course of the winter, suggesting that maintaining residency is beneficial (Duncan 1987; Winter 1986). The Great Gray Owl is the largest owl in North America, and its body size may better enable it to remain on breeding grounds year‐round compared with smaller facultative migrant owls. Smaller owls are less able to withstand food shortages, have higher critical body temperatures, and have more difficulty capturing prey beneath the snow (Mikkola 1983; Korpimäki 1986). When faced with severe locked‐pasture snow conditions, however, even Great Gray Owls responded negatively (via avoidance or long‐distance movement) regardless of whether conditions were associated with increased snow depths, wind crusts, or ice crusts.

The extent to which individuals respond adaptively to variable and extreme limiting conditions likely depends on several factors. Fundamentally, proximate cues that are reliably coupled with environmental conditions are critical for adaptive phenotypic expression (Ghalambor et al. 2007). Shifts in habitat selection and regional movements likely are less feasible for organisms residing in more homogenous systems, such as Arctic tundra or extensive boreal forest, where particularly extensive migrations may be required to escape limiting conditions. For example, a lack of local or regional refuges from limiting conditions may contribute to the continental‐scale irruptions by Great Gray Owls from boreal forests during winter (Collins 1980; Cramp 1985; Nero 1969), whereas owls in more environmentally heterogeneous regions do not exhibit irruptive behavior or extreme long‐distance migrations (Gura and Liston ; Winter 1986). Indeed, facultative migration may be particularly beneficial and prevalent in highly heterogeneous environments (e.g., mountainous regions) (Hahn et al. 2004). Range‐edge populations, such as Great Gray Owls in the GYE, also may be better adapted to respond to environmental variation and extremes compared with core populations, because they tend to experience more extreme environmental conditions, and possess the genetic variation required to cope with such conditions (Rehm et al. 2015). Moreover, individuals in better body condition (Hansen et al. 2019) or with greater dispersal ability or propensity (Claramunt 2021; Steyn, Mitchell, and Terblanche 2016) may be better equipped to use movement to offset stressful conditions. Finally, the range of behaviors across which an organism is plastic may influence responses. Because movement can be energetically costly (Brown et al. 2023), alternative behavioral responses to changing conditions may be preferable when possible. For example, prey‐switching from subnivean prey (e.g., pocket gophers and voles) to species that reside above the snow (e.g., squirrels and birds) may explain how some Great Gray Owls remain in areas of deep snow for the duration of the winter (Bull and Henjum 1990).

Although behavioral plasticity can provide a key buffering mechanism in the face of variable and extreme conditions, plastic individuals nonetheless may incur fitness consequences or trade‐offs (Snell‐Rood 2013; van Buskirk 2012). For example, although evaluating subsequent fitness consequences was beyond the scope of our study, winter snow conditions and resultant behavioral responses potentially influenced Great Gray Owl fitness by altering body condition, timing of arrival on breeding territories, ability to defend breeding territories, reproductive performance, and/or the likelihood of survival. Indeed, although Great Gray Owls avoided wind crusts via proximate shifts in habitat selection, winters with more severe and persistent wind crusts were linked to delayed subsequent nesting and decreased breeding territory occupancy, nest initiation, and number of young fledged (Gura 2023). Furthermore, physiological thresholds certainly exist, beyond which individuals can no longer modulate the effects of limiting conditions via behavior. For example, restrictions on thermal tolerance and acclimation capacity may limit an animal's ability to buffer extreme temperature conditions that change rapidly (Scheffers et al. 2014). Although we reveal mechanistic relationships between snow conditions and facultative movement behavior, we did not investigate potential thresholds (e.g., snow depths and strength of the snow) that govern owl behavior. Great Gray Owls are highly adapted for hunting in snowy landscapes (Clark et al. 2022) and can punch through snow layers strong enough to support the weight of a human (Duncan 1992), yet the specific limits on what snow properties owls can penetrate, and how snow conditions influence foraging success, remain poorly characterized. Future studies that connect facultative behavioral responses with fitness outcomes and identify contextual, physiological limits will be valuable for understanding and predicting species' responses to irregular and rapidly changing environments.

Understanding how organisms cope with environmental unpredictability is a critical priority in ecology, particularly considering the unprecedented ecological changes associated with a changing climate (Malhi et al. 2020). Our work advances such understanding via the evaluation of behavioral responses to increasingly episodic and extreme snow conditions. Moreover, some of the most profound manifestations of a changing climate are occurring in areas of seasonal or long‐term snow (Gulev et al. 2021; Hansen et al. 2010; Liston and Hiemstra 2011). Historically, a lack of synthesis between snow and biological data has restricted research on the effects of the changing cryosphere (Boelman et al. 2019; Reinking et al. 2022). Our use of SnowModel to produce spatiotemporally continuous snow crust data (which, to the best of our knowledge, did not exist prior to our modeling approach) exemplifies how researchers can integrate relevant, scale‐ and process‐specific environmental data with biological information to address persistent, key gaps in ecological understanding. Finally, our finding that the severity and spatiotemporal scale of limiting conditions regulate movement in a facultative migrant sheds light on the key roles of plasticity and resource distribution for the persistence of organisms within a changing world.

## Author Contributions

**Katherine B. Gura:** conceptualization (lead), data curation (lead), formal analysis (lead), funding acquisition (equal), investigation (lead), methodology (equal), project administration (equal), visualization (lead), writing – original draft (lead), writing – review and editing (lead). **Glen E. Liston:** conceptualization (equal), data curation (lead), formal analysis (supporting), investigation (supporting), methodology (equal), resources (supporting), software (lead), supervision (supporting), writing – review and editing (equal). **Adele K. Reinking:** conceptualization (supporting), data curation (supporting), investigation (supporting), methodology (supporting), resources (supporting), software (supporting), writing – review and editing (equal). **Bryan Bedrosian:** conceptualization (supporting), funding acquisition (equal), investigation (supporting), project administration (supporting), resources (equal), supervision (supporting), writing – review and editing (equal). **Kelly Elder:** conceptualization (supporting), investigation (supporting), methodology (supporting), writing – review and editing (equal). **Anna D. Chalfoun:** conceptualization (supporting), formal analysis (supporting), funding acquisition (equal), investigation (supporting), methodology (supporting), project administration (lead), resources (equal), supervision (lead), writing – original draft (supporting), writing – review and editing (equal).

## Conflicts of Interest

The authors declare no conflicts of interest.

## Data Availability

Data underlying this manuscript currently are archived and publicly available at Dryad, an open access data repository (http://datadryad.org/stash/share/Ow9Ebkkg_4PIhcKP0fa9HvYzCyByuTWplxI9d0r0LkI) (Gura and Liston 2024).

## Appendix A

**TABLE A1:** Top models of habitat selection at the daily step level by adult Great Gray Owls (*n* = 42) in the Greater Yellowstone Ecosystem during the winters of 2017–2022.

Model	*K*	*n*_event_	QIC	Quasi‐LL
CWCS + SD	3	2936	10315.05	−5150.804
CWCS + WCES + SD	4	2936	10315.74	−5150.248
CWCS + ICP + WCES + SD	5	2936	10316.92	−5149.734
CWCS + ICES + ICP + WCES + SD	6	2936	10317.42	−5149.199
CICS + CWCS + ICES + ICP + WCES + SD	7	2936	10319.75	−5149.136
CWCS	2	2936	10347.90	−5168.085
Null	1	2936	10530.10	−5260.411

*Note:* Models were based on population‐level averages of integrated step selection analysis of the endpoints of daily steps. Model parameters include cumulative wind crust severity (CWCS), wind crust event severity (WCES), snow depth (SD), ice crust event severity (ICES), and ice crust persistence (ICP). “*K*” indicates number of parameters in the model, “*n*_event_” indicates the number of movement steps used to fit the model, “QIC” indicates the Quasi‐likelihood Information Criteria value, and “QuasiLL” indicates the quasi‐likelihood of the model.

**TABLE A2:** Top models summarizing environmental covariates influencing the probability of winter long‐distance movements by adult Great Gray Owls (*n* = 42) in the Greater Yellowstone Ecosystem during the winters of 2017–2022.

Model	*K*	AICc	ΔAICc
ICESPW + SD + WCESPW	3	897.772	0
ICESPW + SD + WCES + WCESPW	4	898.702	0.929
ICES + ICESPW + SD + WCES + WCESPW	5	899.522	1.750
CWCS + ICES + ICESPW + SD + WCES + WCESPW	6	900.893	3.121
CWCS + CICS + ICES + ICESPW + SD + WCES + WCESPW	7	902.470	4.698
CWCS + CICS + ICES + ICESPW + SD + WCES + WCESPW + WCP	8	904.056	6.284
CWCS + CICS + ICES + ICESPW + ICP + SD + WCES + WCESPW + WCP	9	905.860	8.087

*Note:* Models are based on Cox proportional hazards analysis of the probability of a long‐distance movement in response to ice crust persistence (ICP), ice crust event severity (ICES), ice crust event severity the prior week (ICESPW), cumulative ice crust severity (CICS), cumulative wind crust severity (CWCS), snow depth (SD), wind crust persistence (WCP), wind crust event severity (WCES), and wind crust event severity the prior week (WCESPW). “*K*” indicates number of parameters in the model, “AICc” is the small‐sample corrected Akaike Information Criterion value, and “ΔAICc” refers to the change in Akaike's Information Criterion value adjusted for small sample size.
